# S09 Exploring the sport and physical activity experiences of people with disabilities and members of the LGBTQ+ community in Ireland: Breaking down barriers, building new relationships

**DOI:** 10.1093/eurpub/ckae114.235

**Published:** 2024-09-26

**Authors:** Benny Cullen

**Affiliations:** Sport Ireland, Dublin, Ireland

## Abstract

Sport Ireland (SI) will deliver a symposium that will provide valuable insights on the importance of inclusive sport and physical activity environments and opportunities that cater for the needs of all in society. The symposium will share results from a selection of studies funded by the SI Research Grant Scheme (RGS), which has funded 49 projects across 13 universities since its inception in 2021. The RGS focuses on projects that address issues of inclusion in sport and physical activity with a view to empowering marginalised communities. The symposium will bring together the lead investigators from four such projects, who seek to impact their chosen field of study while implementing programmes that will initiate change in both policy and the lives of those they engage with.

Led by Robert Purcell, Active Disability Ireland’s study explores the experiences of young people with disabilities in Ireland; including barriers to physical activity, parent/guardian perceptions of need and lack of available supports / facilities.

Lisa Flynn, Vision Sports Ireland, will provide new insights on physical activity experience for children and adults with a visual impairment. The study focuses on children who attend mainstream schools and explores the experiences of their PE teachers. This programme is an ongoing collaboration between Vision Sports Ireland, the Department of Education and DCU.

Dr. Hayley Kavanagh from Special Olympics Ireland and DCU will present findings from a study on fundamental movement skills (FMS) in children with an intellectual disability and the development of a new coaching programme to address the many issues uncovered.

Mr Seamus Nugent, Sport Inclusion lead at Kilkenny Recreation & Sport Partnership, will present findings from ‘Equality in the Field’, a study that examines the experience of LGBTQ+ Youth participation in sport.

The symposium will be chaired by Mr Benny Cullen, the Director of Sport and Innovation Unit at Sport Ireland. Mr Cullen has led on several largescale population studies, including the Irish Sport Monitor and Children’s Sport & Physical Activity study. Mr Cullen will start the symposium with a brief introduction of Sport and Physical Activity policy in Ireland as it relates to diversity and inclusion.

